# Effects of Topical 1% Sodium Hyaluronate and Hydroxypropyl Methylcellulose in Treatment of Corneal Epithelial Defects

**Published:** 2016

**Authors:** Kourosh SHAHRAKI, Seyed-Rafi HOSSEINI, Atefeh AMINI FARD, Hashem SHADEMAN, Kianoush SHAHRAKI, Amir Masood SALARI, Mohammad-Naeim AMINI FARD

**Affiliations:** 1 Department of Ophthalmology, Alzahra Eye Hospital, Zahedan University of Medical Sciences, Zahedan, Iran; 2 Department of Optometry, School of Rehabilitation, Zahedan University of Medical Sciences, Zahedan, Iran; 3 Food Science and Industry, Khozestan Sciences and Research Branch, Islamic Azad University, Ahvaz, Iran; 4 Department of Ophthalmology, Farabi Eye Hospital, Tehran University of Medical Sciences, Tehran, Iran

**Keywords:** Sodium Hyaluronate, Hydroxypropyl Methylcellulose, Corneal Epithelial Defects, Healon, HPMC

## Abstract

We aimed to compare the therapeutic effects of topical 1% sodium hyaluronate (Healon) or hydroxypropyl methylcellulose (HPMC) for the treatment of alkali-induced epithelial corneal defects. An alkali burn was produced in 30 corneas of 30 New Zealand White rabbits, using a 7.5-mm-diameter trephine. The rabbits were randomly divided into three groups. Four times a day, one group was treated with 1% sodium hyaluronate, one with HPMC, and one (the control group) with physiologic saline. During the treatment period, the size of the epithelial defect was observed every day, up to day 17, using a slit-lamp biomicroscope (with fluorescein). Sodium hyaluronate significantly accelerated the wound healing process compared with saline and increased the healing rate to an even greater extent compared with HPMC. Sodium hyaluronate, but not HPMC, is an effective wound-healing adjuvant for alkali-induced corneal epithelial defects.

## INTRODUCTION

The cornea is a transparent and flat layer of the eye that allows light to pass through to reach the inside of the eye. The transparent corneal epithelium prevents any damage to the lower layers of the cornea by acting as a barrier against microorganisms. Any damage to this layer may affect its transparency and reduce its protective capacity (1, 2). Corneal epithelial defects are a relatively common ocular condition, and attempts to treat these defects are considered to be a major goal of ophthalmic research. The mechanism for repairing and rebuilding the corneal epithelium is very similar to the mechanism involved in other mucosal layers, and it includes the migration and proliferation of epithelial cells. It is believed that when damage to the epithelium occurs, limbal stem cells migrate towards the center of the damage and proliferate rapidly, thereby filling the damaged area with new cells (3-5). Various factors can influence the healing of epithelial defects, including the presence of growth factors, umbilical cord serum, and autologous serum (6-10), the cell migration rate, and the rate of desmosome production between the epithelial cells. Sodium hyaluronate is a viscoelastic substance that is widely used to treat ocular conditions. It is a mucopolysaccharide found naturally in vitreous humor and synovial fluid, where it acts as a “tissue cement” (11-15). Hydroxypropyl methylcellulose (HPMC) is also a viscoelastic polymer that can be synthesized from cellulose. It has been proposed that sodium hyaluronate may be a useful treatment for epithelial defects because it increases cell migration and the number of hemidesmosomes (which attach cells to the extracellular matrix). Several studies have been conducted in this area, but none has decisively confirmed this theory (16-19).

A 2004 study by Gomes et al. showed that human corneal epithelial cell in sodium hyaluronate-enriched medium exhibited significantly increased migration compared with cells in HPMC-enriched medium. Promotion of cell migration can subsequently lead to rapid wound closure (8). In addition, a 2013 study by Wu et al. showed that sodium hyaluronate can protect corneal epithelial cells from alkali burns and promote healing of corneal epithelial tissue (7). The authors also showed that the molecular weight of sodium hyaluronate is a crucial factor in determining its effects, and only high-molecular-weight sodium hyaluronate significantly reduced NaOH-induced cytotoxic effects in corneal cells and increased their migration and wound healing ability (7). A recent study by Zhong et al. evaluated the effects and mechanism of action of sodium hyaluronate in the promotion of corneal epithelial wound healing, and it showed that sodium hyaluronate enhanced wound healing by promoting cell migration, upregulating repair responses, and suppressing inflammatory responses (14). In 1998, in a similar study in South Korea, the use of 1% sodium hyaluronate (Healon) four times a day to treat a 5.5-mm-diameter corneal epithelial defect was shown to promote a higher recovery rate compared to the control treatment (phosphate-buffered saline). This is likely to have been due to the higher number of hemidesmosomes bonds involving the basement membrane layer (14, 15). Thus, the present study was designed to test whether 1% sodium hyaluronate could be a useful treatment for epithelial defects. 

## MATERIALS AND METHODS

The study involved 30 homogeneous rabbits (New Zealand White rabbits of the same aged 6 months, weighing 2.0–2.5 kg, which were provided by the Razi Institute, Iran). The rabbits were randomly divided into three groups, each consisting of 10 rabbits. Corneal alkali burns were produced in accordance with the method devised by Ormerod et al., with some modifications that have been described previously, which are described below (16). Briefly, the study was conducted in accordance to the principles of double-blind clinical trial, and the rabbits were injected intramuscularly with sedatives (37.5 mg kg^-1^ ketamine and 10 mg kg^-1^ xylazine). Local anesthesia was achieved using 0.5% tetracaine eyedrops (SinaDarou, Tehran, Iran Anestocaine®). Subsequently, an 8-mm epithelial defect was produced at the center of the left cornea of each rabbit using a 7.5-mm-diameter trephine along with 0.4 mL of 1 M NaOH for 35 s. The damaged site was then rinsed with 20 mL of 0.9 M saline. As the study was conducted in sterile conditions, there was no need to use antibiotic drops. To decrease bias, we excluded rabbits with defects > 8 mm and those with an unusual pattern of corneal surface disruption (such as infection or perforation) before starting the treatment and more rabbits added to each group up to 10. The treatments and assessments of the defects began the following day. Four times a day, one group was treated with 1% sodium hyaluronate, one was treated with HPMC, and the control group was treated with physiologic saline. The treatments continued until the epithelial defects were completely healed. The researcher who administered the treatments and the researcher in charge of assessing the defects in the rabbits were both blinded to the treatment groups (i.e., the treatments were labelled with numbers rather than their names).

 We used a slit-lamp biomicroscope (HAAG-STREIT AG, Bern, Switzerland) to assess the defects. Fluorescein (an orange dye) was used in order to ensure that stimulation of the cornea and conjunctiva and epithelial infections could be easily discerned. The decrement of the defect diameter and corneal improvement was measured during the assessments. To measure the size of each defect, the slit-lamp biomicroscope was used to measure the largest diameter of the defect (i.e., when the width of light from the device became increasingly thin slit size) in millimeters. 

**Figure 1:**
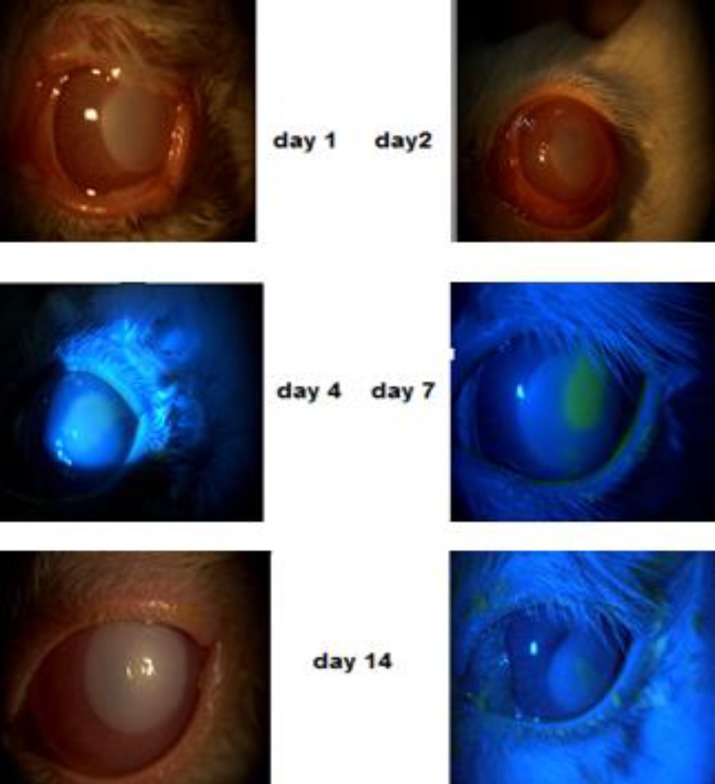
Slit-lamp photographs of the cornea of a representative rabbit in the group treated with 1% topical sodium hyaluronate (Healon) on days 1, 2, 4, 7, and 14

Each rabbit was checked once a day until the defect was completely healed. To provide representative documentation of the healing process, one rabbit was chosen from each group, and four color photographs of the epithelial defect were taken on days 1, 2, 4, and 7 during the treatment, as shown in Figure 1 for the rabbits in the sodium hyaluronate group. After each assessment, the data were recorded. SPSS version 16 (SPSS Inc., Chicago, IL, USA) was used to analyze the data. The between-group differences in the mean sizes and the number of days to compete recovery were tested using XYZ tests, and P values < 0.05 were considered significant. At the end of the experiment, the treatment groups were disclosed, and the results were interpreted.

This study was conducted in accordance with the provisions set out in the Statement for the Use of Animals in Ophthalmic and Visual Research from the Association for Research in Vision and Ophthalmology, and with respect to the care committee and animal use in Ophthalmology Research (ethical approval number 426_T). The techniques used were selected in order to cause the least amount of pain (and other stimulation) possible to the animals. Before injuring each cornea, we anesthetized the area using tetracaine. If an assessment was difficult due to a rabbit’s movement, the rabbit was injected with 37.5 mg kg^-1^ ketamine. Only one eye of each animal was injured, so as to disrupt their natural lives as little as possible.

## RESULTS

The following mean epithelial defect diameters (*± *SD) were measured on day 1: 39 ± 7.46 mm^2^ for the sodium hyaluronate group, 37.1 ± 6.45 mm^2^ for the HPMC group, and 39.1 ± 8.17 for the control group. There were not significant differences in the sizes on day 1 (P = 0.794) (Table 1).

On day 2, there was a significant reduction in the mean diameter of the epithelial defects compared to day 1 (in both statistical and clinical terms) for all groups (P < 0.05), to 15 ± 1.12, 16.5 ± 7.62, and 23.8 ± 1.47 mm^2 ^for the groups treated with sodium hyaluronate, HPMC, and saline, respectively. However, even on day 2, the between-group differences in the size of epithelial defects were not significant (P = 0.21) (Table 1).

**Figure 2:**
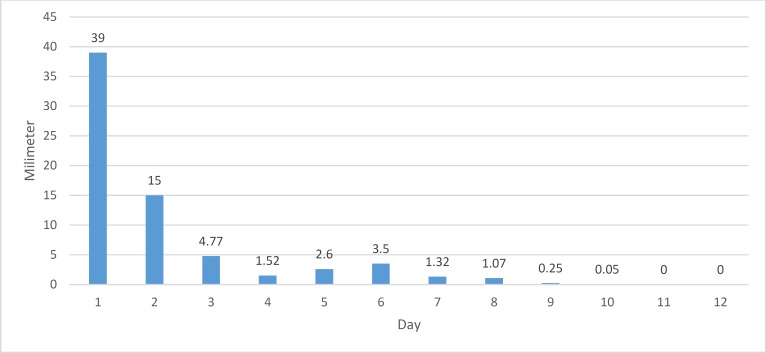
Size of corneal epithelial defect by day of treatment in the 1% sodium hyaluronate (Healon) group

As shown in Figure 2, the improvement in the rabbits in the sodium hyaluronate group increased considerably after day 2, though minor increases in the size of the epithelial defect were observed on days 5 and 6. Full recovery was observed in this group on day 11 (Table 2).

**Table 1:** Comparison of the size of the corneal epithelial defects in all three groups on day 1 to 8 of treatment

	**Days**							
**Groups**	**1**	**2**	**3**	**4**	**5**	**6**	**7**	**8**
**1% sodium hyaluronate (Healon)**								
Mean	39.0	15.0	4.78	1.52	2.6	3.52	1.32	1.07
SD	7.468	1.127	4.004	2.44	2.63	3.24	1.22	1.65
**HPMC**								
Mean	37.1	16.5	18.0	13.2	5.6	4.4	3.9	5.15
SD	6.454	7.619	8.47	7.06	3.89	2.5	3.66	9.092
**Control (saline)**								
Mean	39.1	23.8	21.0	19.9	15.4	10.6	12.2	15.4
SD	8.17	1.47	7.19	4.75	6.83	5.168	7.004	1.265
**P value**	0.8	0.2	0.0001	0.0001	0.0001	0.0001	0.0001	0.004

HPMC, hydroxypropyl methylcellulose; SD: standard deviation

**Figure 3:**
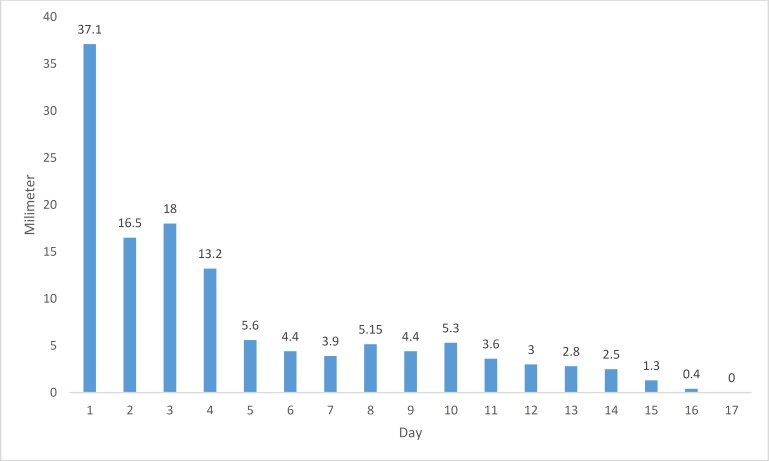
Size of corneal epithelial defect by day of treatment in HPMC group

As shown in Figure 3, there was a relatively steady decrease in the size of the epithelial defect in the HPMC group, though minor increases in the size were observed on days 8 and 10. However, there was quick recovery from day 11 (Table 2). Full recovery was observed by day 17 (Table 2). Despite the difference in the rate of healing between the groups, the degree of corneal opacity was almost the same after complete healing of the defects.

**Figure 4:**
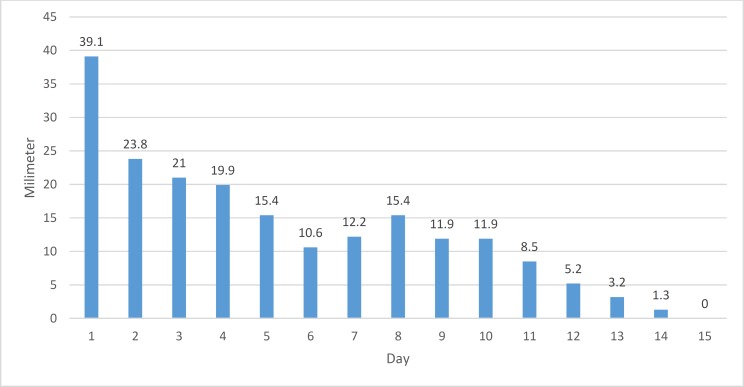
Size of corneal epithelial defect over time in the control group

**Figure 5:**
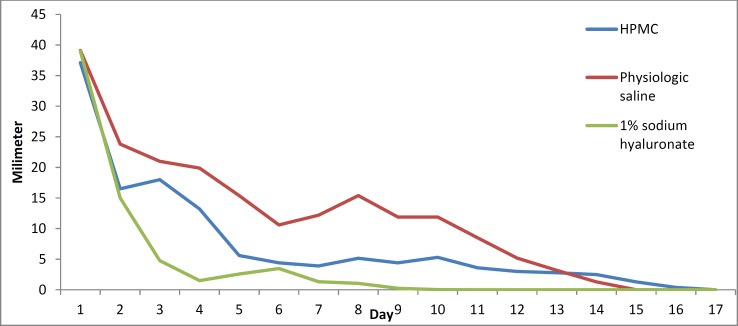
Comparison of the size of corneal epithelial defects in all three groups by day of treatment

The results for the control group are shown in Figure 4. There was a relatively steady recovery, with minor increases in the size of the epithelial defect on days 7 and 8, which improved quickly thereafter. Full recovery occurred by day 15 (Table 2). Figure 5 shows a between-group comparison of the improvement in the size of the corneal epithelial defects over time.

**Table 2:** Comparison of the size of the corneal epithelial defects in all three groups on day 8 to 17 of treatment

**Group**	**Day**								
	**9**	**10**	**11**	**12**	**13**	**14**	**15**	**16**	**17**
**1% sodium hyaluronate (Healon)**									
Mean	0.25	0.05	0	0	0	0	0	0	0
SD	0.408	0.105	0	0	0	0	0	0	0
**HPMC**									
Mean	4.4	5.3	3.6	3.0	2.8	2.5	1.3	0.4	0
SD	7.5	8.92	5.93	6.13	1.3	1.22	0	0	0
**Control (saline)**									
Mean	11.9	11.9	8.5	7.9	3.2	1.3	0	0	0
SD	1.04	1.3	1.027	1.078	1.54	1.1	0	0	0
**P value**	0.006	0.025	0.033	0.061	0.64	0.32	0.001	0.01	0

HPMC, hydroxypropyl methylcellulose; SD: standard deviation

HPMC, hydroxypropyl methylcellulose

HPMC, hydroxypropyl methylcellulose

## DISCUSSION

This study was conducted in accordance with the principles of double-blind clinical trials, and it involved the use of a trephine to make identical epithelial defects in order to prevent potential biases between the three treatment groups (i.e., there was no significant between-group difference in the epithelial defects at the beginning of the experiment). Corneal epithelial defects are common in both industrial and non-industrial societies, and patients with this condition constitute a considerable proportion of cases presenting at optometrists’ clinics. Shortening the treatment duration for patients with epithelial defects (irrespective of the causes of the wounds) would result in less morbidity; a lower likelihood of side effects associated with the wound, and increased quality of life. It would also reduce the productivity losses caused by the long recuperation period for patients, their families, wider society, employers, and insurance companies. Therefore, it is clear that developing treatments that shorten the treatment duration is a priority for medical researchers and health services. Reducing the treatment duration is particularly important for patients with corneal epithelial damage because, as the cornea has no blood vessels, it has less potential for recovery compared to other tissues. This further leads to an increased risk of infection, development of corneal opacity, and scarring if the cornea is subject to long-term epithelial defects, which can result in permanent visual impairment or blindness. In addition to its important role in refraction, the cornea also acts as one of the most important protective barriers in the visual system, so increasing the rate of recovery from corneal damage, even by one day, is of great importance (9, 17-22).

The epithelial defects in the rabbits treated with sodium hyaluronate were completely healed in < 11 days. The results of this study concur with those of a study by Wu CL et al., which concluded that 1% sodium hyaluronate increased the epithelial defect recovery rate (7). However, interestingly, in a 1989 study in Sweden by Chung et al 1% and 2% sodium hyaluronate did not decrease the epithelial defect recovery rate (9). The results of our study and the study by Wu CL et al. appear to support those of studies such as a study by Gomes et al. (8) and a study in Israel by Steiebel-Kalish based on animal models (10). It appears that the high recovery rates might be the result of fewer inflammatory cells (such as polymorphonuclear cells) in the corneal stroma and more keratocysts. As the results indicate, although the rabbits treated with HPMC and saline fully recovered, the treatment duration in the HPMC group was 17 days, and that in the control group was 15 days. In comparison, the treatment duration in the sodium hyaluronate group was 11 days, and the between-group differences were statistically significant. Although 1% sodium hyaluronate has one-fourth the volume and three times the cost of HPMC, it appears to be a useful treatment for corneal epithelial defects, as it statistically and clinically significantly reduced the treatment duration in rabbits. In addition, 1% sodium hyaluronate may be especially useful for treating patients for whom treatments with artificial tears (such as HPMC-based eyedrops) and saline have failed. The fact that the treatment duration with HPMC is longer than that associated with saline indicates that HPMC should not be used to treat corneal epithelial defects.
